# Usability and Acceptability of an App-Based Approach to Treat Low Back Pain: Preplanned Secondary Analysis of a Randomized Controlled Trial

**DOI:** 10.2196/59866

**Published:** 2025-08-25

**Authors:** Svenja Kaczorowski, Katharina Trompeter, Carolin Kramer, Christian Grüneberg, Christian Thiel, Lars Donath, Daniel L Belavy

**Affiliations:** 1 Department of Intervention Research in Exercise Training, German Sport University Cologne, Cologne, Germany; 2 Division of Physiotherapy, Department of Nursing, Midwifery and Therapeutic Sciences, Bochum University of Applied Sciences, Gesundheitscampus 6-8, Bochum, 44801, Germany, 49 23477727776

**Keywords:** telemedicine, mHealth, low back pain, app-based intervention, usability, acceptability, app, randomized controlled trial, patient compliance, app usage, pain, mobile phone

## Abstract

**Background:**

Low back pain (LBP) is a major cause of disability worldwide. To tackle issues such as long wait times and limited access to conventional care, telemedicine is emerging as a viable alternative. It offers benefits such as reduced travel and increased flexibility, with evidence showing comparable effectiveness to in-person care. However, usability remains a key challenge, impacting patient compliance.

**Objectives:**

In a 3-arm randomized controlled trial, our preplanned secondary analysis aimed to assess the usability and patient perceptions toward an autonomous app-based intervention (“NOLA”) for LBP to improve clinical practice of telemedicine interventions. Objectives included evaluating app usability, assessing perceptions toward telemedicine, and exploring app usage, adherence, and motivation.

**Methods:**

Patients with LBP were recruited from May to August 2022 and randomized into App, Physio+App, or Physio groups. App and Physio+App groups were included in this subanalysis. Intervention duration was 6 weeks. Data on baseline characteristics, System Usability Scale, Telemedicine Perception Questionnaire, app usage, adherence, and motivation were collected via web-based questionnaires.

**Results:**

A total of 64 participants were randomized to use the app with available data for 38 participants. The mean age of participants who completed was 49.9 (SD 13.6) years, with 78% (29/38) experiencing LBP for more than 2 years. Usability scores (0‐100) were good (Physio+App: median 78, IQR 58-92, app: median 86, IQR 65-91). Positive telemedicine perceptions were noted, with 84% (15/20) rating it an adequate addition to usual care. App usage varied, with 43% (16/35) using it 3 to 5 days per week, and 64% (21/33) reported motivation to use the app. The dropout rates (App: 14/32, 44%; Physio+App: 12/32, 38%; Physio: 11/29, 38%) were similar, but participants who dropped out had statistically significantly less pain (completers: mean 3.9, SD 2.0; dropouts: mean 3.0, SD 2.0; *P*=.02). Reasons for dropout were mostly not reported.

**Conclusions:**

The app “NOLA” demonstrated good usability, and participants expressed positive perceptions toward telemedicine in those who completed the intervention. Despite concerns about the lack of physical contact, the majority considered telemedicine a convenient form of health care delivery. App usage and motivation were favorable, emphasizing the potential of app-based interventions in managing LBP.

## Introduction

Low back pain (LBP) is the most common cause for disability worldwide [1] with a high economic burden of health care costs, especially in developed countries [2], and creates up to US $108 billion of health care costs per year [3]. Recommendations for the treatment of patients with LBP include an active approach that also takes education into account [4]. Treatment of patients with LBP usually takes place in an in-person setting. Waiting times, insufficient infrastructure, the recent COVID-19 pandemic, and access to therapy emphasize the need for the integration of alternative approaches into LBP care. The importance of telemedicine as an alternative or adjunctive to conventional physiotherapy has increased rapidly in the last decade [5,6]. Telemedicine shows several advantages compared to standard in-person care, such as reduced travel times [7], increased time flexibility [8,9], and self-management [10]. Several pathways exist to deliver telemedicine, such as websites, apps, videoconferencing, or phone calls. The current evidence shows that telemedicine tends to be effective for patients with LBP in improving pain, physical function, and quality of life compared to conventional care [11,12].

While the effectiveness of an intervention is an important factor, it is also essential to consider patients’ perceptions and attitudes toward the intervention. With the increasing use of telemedicine, it became clear that it is generally accepted by patients seeking help for musculoskeletal conditions [13-15]. However, a recent umbrella review revealed that 1 main barrier in the implementation of telemedicine is the usability of a digital intervention, which is linked to adherence to the intervention [16]. Due to a variety of possibilities for how telemedicine can be delivered, factors that influence usability are specific for each mode of delivery [17]. The systematic review of Thurnheer et al [18] showed that mobile apps in pain management have, in general, good usability, but details that improve usability remain unclear. To support the implementation of mobile apps into clinical practice, it is of crucial importance to investigate factors that influence acceptance and usability.

This paper aimed to evaluate the usability of the “NOLA” app, which integrates exercise and education for patients with LBP, as well as patient perceptions of telemedicine in patients who received an app-only intervention compared to those who received in-person physiotherapy in addition to the app. Further, this study aimed to evaluate the frequency of use of the app, compliance with the telemedicine intervention, and participants’ motivation to use the app. These factors are important in shaping future clinical trials and improving the current clinical practice of telemedicine interventions to enhance patient satisfaction.

## Methods

### Study Design

This study is a preplanned secondary analysis of a 3-arm parallel randomized controlled trial. The main analysis on effectiveness, adherence, and sustainability is to be reported elsewhere by the principal investigator. The trial has been conducted per the Declaration of Helsinki and followed recommendations of the CONSORT (Consolidated Standards of Reporting Trials)–eHealth checklist [19]. The trial took place in 2 physiotherapy practices in the Ruhr area in Germany.

### Ethical Considerations

The trial was approved by the local ethical committee of the Hochschule für Gesundheit (University of Applied Sciences; ID: 220317) and registered at the German Clinical Trial Registry (DRKS00029099). Participants who consented to participate in the main trial agreed to the use of their data for this secondary analysis. Data have been anonymized for the analysis. The participants did not receive any direct financial compensation but could use the app for free during the time of this trial.

### Patient and Public Involvement Statement

No patient or public involvement occurred in this study.

### Recruitment

Participants were recruited from 9 orthopedic practices in the Ruhr area in Germany from May to August 2022. Additionally, recruitment was done via social media (Instagram [Instagram from Meta] and Facebook [Meta]) and local newspaper advertisements. In case of interest, potential participants were contacted via telephone and informed about the benefits and risks of this study. If they were still interested, they were invited to 1 of this study’s sites to review the eligibility for this study. All participants signed an informed consent document before being randomized into 1 of the 3 groups. To control for expectation bias, it was explained to the participants that it was unknown which intervention was superior. This study presents a preplanned exploratory analysis of secondary outcomes of a randomized controlled trial. The sample size was determined for the preplanned primary outcome “pain intensity” of the main trial, which is not included in the present analysis. Formal sample size calculations were not performed for the current secondary outcomes to avoid unnecessarily inflating this trial’s size.

### Eligibility Criteria

Individuals older than 18 years were included if they had a diagnosis of LBP (*ICD-10* [*International Statistical Classification of Diseases, Tenth Revision*] M54.5) with pain lasting for at least 6 weeks. Participants were required to have access to a smartphone or tablet and be able to use it. Further inclusion criteria were sufficient knowledge of German, willingness to travel to the treatment centers, and cognitive capacity to understand this study’s information and give informed consent. Exclusion criteria included patients with LBP originating from a serious pathology (eg, fractures, spinal stenosis, or tumors), patients with complaints in other body parts that made participation in exercise training not feasible, and patients who were pregnant.

### Randomization and Blinding

Participants were randomized using permuted block randomization with random block sizes. A randomization list was generated online (Sealed Envelope Ltd 2021, 2022) with only subject ID assignment. The allocation ratio between groups was 1:1:1. Randomization was performed after clinical baseline tests for the main analysis were completed. Group allocation was communicated verbally to the participants, as this allowed any unanswered questions about the respective intervention to be discussed directly. Participants and physiotherapists could not be blinded to randomization because they were performing and administering the active intervention.

### Intervention

#### Overview

Participants were randomized into 1 of the following 3 groups: App, Physio+App, or Physio. Participants in the Physio+App group also received access to the app “NOLA” in addition to standard physiotherapy. Only the first 2 groups (App, Physio+App) were included in this preplanned secondary analysis. The intervention duration was 6 weeks for both groups.

#### App Intervention

**Figure 1.**
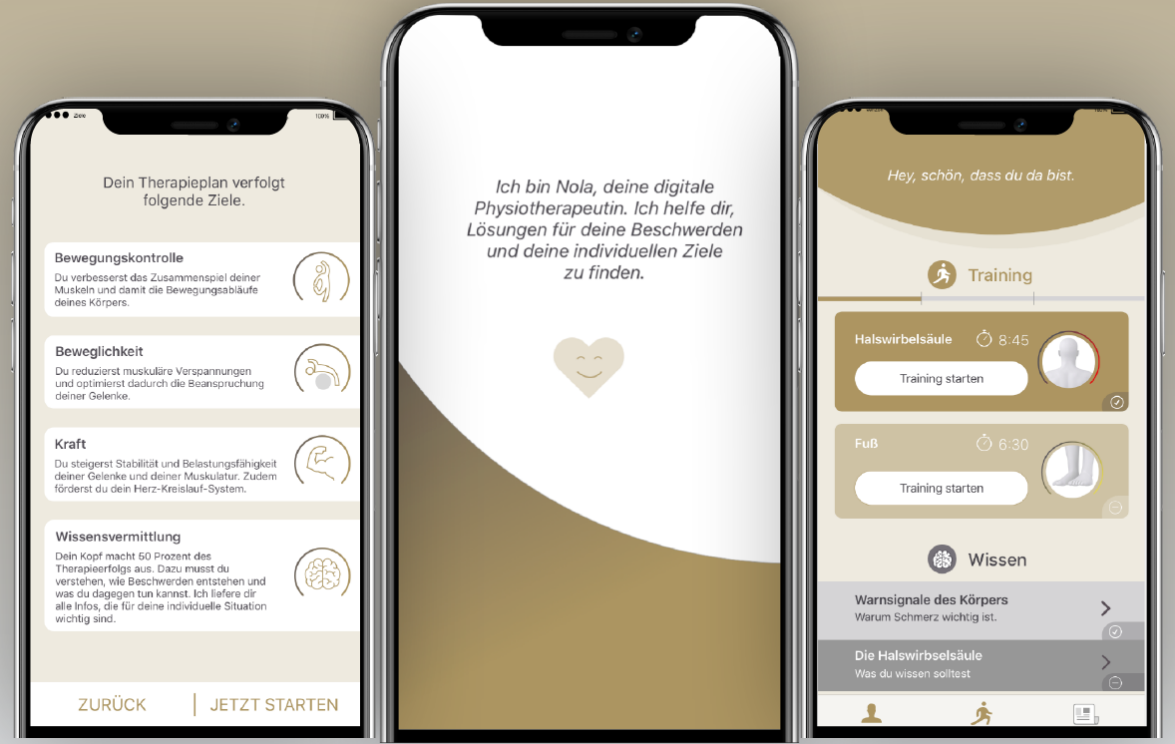
Screenshots of the NOLA app that participants in both included groups used on their mobile devices. The app contains an exercise program as well as educational content.

Participants in the App group were given access to the app “NOLA” (MyLetics GmbH), which has been developed by physiotherapists, medical doctors, and information technology experts. It is stand-alone software per the European Medical Device Directive MDD (2007/47/EC), which can be set up on the smartphone without any additional devices (Figure 1). The training program integrated in Nola can be used autonomously. First, each user completes an anamnesis in which questions about age, sex, current complaints, and treatment goals are answered. The anamnesis takes into account medical, pain-physiological, and training-scientific parameters. On this basis, a personalized therapy and training plan is drawn up automatically, which is adapted to the individual’s training progress. In addition to training, the multimodal therapy plan includes everyday exercises and knowledge lessons. On the day of the initial medical examination, a short introduction to the app was provided. Participants were advised to complete a minimum of 2 sessions per week, while no maximum threshold was set. Reminders to exercise were sent out via push messages regularly. Each session ranged from 8 to 25 minutes, depending on the current pain status, and contained exercises that were demonstrated in video tutorials and could be performed without additional equipment.

#### Physiotherapy Intervention

Participants received the physiotherapy intervention in the practice “Schwerpunktpraxis für Human Movement” in Essen, Germany. The content of the physiotherapy intervention was at the discretion of the therapist and consisted of one or more of the following: manual therapy, strength training, stabilization exercises, or stretching. The app usage was integrated into the therapeutic process so that exercises could be explained and supervised directly by the therapist. All 4 physiotherapists were trained and experienced in the integration of the app in the therapeutic process. A standardized introduction to the use of the app was achieved through internal training of the physiotherapists. Participants were advised to complete a minimum of 2 sessions per week, including the physiotherapy session. To reflect the reality of care in Germany, it was possible for participants to attend 6 to 12 physiotherapy sessions in person overall with a duration of 20 minutes each.

### Outcomes

Baseline characteristics are reported for demographic variables (sex, age, height, and weight), pain duration, medication use, and current sick leave, as well as for pain intensity (numeric rating scale: 0‐10) and disability (German Oswestry Disability Index: 0‐32 [20]). For this preplanned secondary analysis, we collected data on the usability of the NOLA app and perceptions of patients toward telemedicine in general. We used a German translation of the System Usability Scale [21] (SUS) and adapted the wording accordingly to the use of an app (Multimedia Appendix 1). The overall score ranges from 0 (worst) to 100 (best). Further, we used the German translation of the Telemedicine Perception Questionnaire [22] (TMPQ) and adapted it to only use meaningful questions regarding the app-based intervention (Multimedia Appendix 2). As we only used a selection of questions from the TMPQ, the overall score ranged from 0 (worst) to 60 (best). Furthermore, the self-reported frequency of app usage, the adherence, motivation, and perceptions toward the future use of the app were evaluated by multiple-choice questions, which were piloted before data collection started. In case of a dropout after the start of the intervention, we asked for the reasons for the dropout. All data used in our analysis were collected online via “SoSci Survey” (SoSci Survey GmbH) at the end of the intervention. There were no changes to trial outcomes after this trial commenced.

### Deviations From the Registered Trial Protocol

We recruited fewer participants than planned for the main analysis on pain intensity. Importantly, this secondary analysis was not part of the original power calculation and should therefore be considered exploratory in nature.

### Statistical Analyses

Frequencies of answers for each question in the SUS, TMPQ, adherence, and motivation questionnaire were calculated for each group, as well as the overall score as median and its IQR for the SUS and TMPQ. Between-group differences of the overall scores have been compared with the Wilcoxon rank sum test. To evaluate a potential association between the improvement in pain intensity and the TMPQ score, we used a simple linear regression model. Baseline demographics (age, pain intensity, and disability) have been compared between those who dropped out and those who completed the intervention with the Wilcoxon rank sum test. The level of statistical significance was set to 5% for all analyses. The analysis has been conducted with RStudio (R Foundation, version 4.3.1). The code is available on Open Science Framework [23].

## Results

### Patient Characteristics

**Figure 2.**
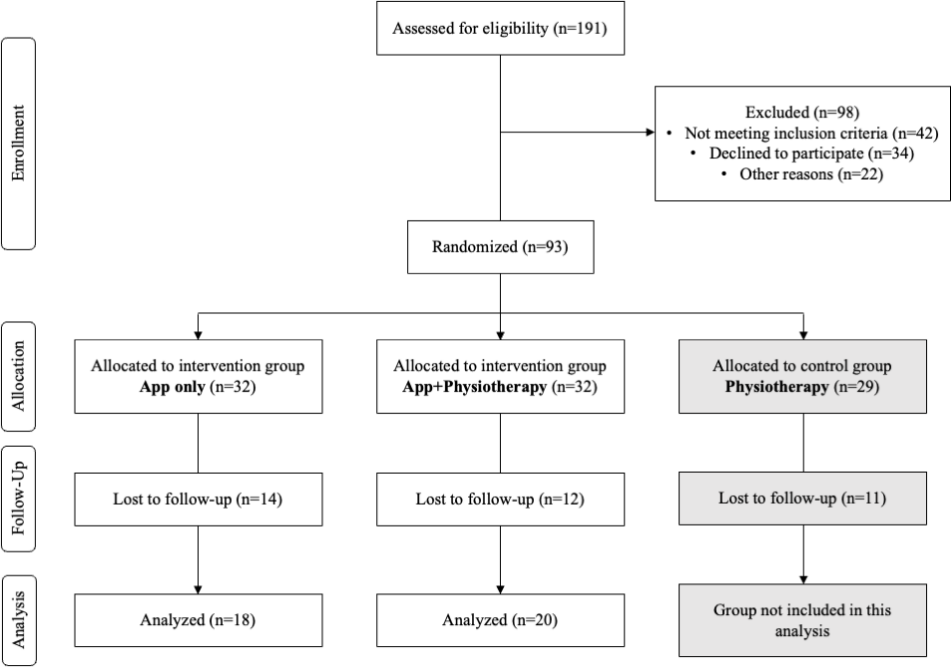
CONSORT flow diagram showing the flow of participants through this study. Only the “App only” and the “App+Physiotherapy” groups are included in this secondary analysis. The “Physiotherapy” group is included in the main analysis. The analysis was conducted based on outcomes that were collected at the end of the intervention. CONSORT: Consolidated Standards of Reporting Trials.

Of 191 initially screened potential participants, 64 were randomized equally into the Physio+App and App group. Additionally, 29 participants were randomized into the Physio group, which was not included in this secondary analysis. From those randomized, 20 participants (12 female) from the Physio+App group and 18 (11 female) from the App group were followed up and included in this analysis. Figure 2 shows a flowchart of the enrollment process.

The mean age of all participants from those 2 groups who completed was 49.9 (SD 13.6) years. Most of the participants (29/38, 78%) had LBP for more than 2 years. Of the 38 participants, 15 (39%) participants currently used pain medication and 7 (18%) were on sick leave. The average pain intensity at baseline was 3.9 (SD 2.1) and the mean Oswestry Disability Index score 24.6 (SD 9.6). Baseline characteristics for each group are displayed in Table 1.

**Table 1.** Baseline characteristics obtained from the sample that was included in the secondary analysis on usability and acceptability of an app-based intervention. All data were self-reported and collected in web-based questionnaires.

Variable	Overall, N=38	App only, n=18	Physio+App, n=20
Gender, n (%)			
Female	23 (61)	11 (61)	12 (60)
Male	15 (39)	7 (39)	8 (40)
Age, mean (SD)	49.87 (13.55)	51.83 (14.09)	48.10 (13.15)
Height, mean (SD)	174.79 (10.59)	172.72 (10.09)	176.65 (10.95)
Weight, mean (SD)	84.34 (20.63)	82.28 (20.56)	86.20 (21.05)
Pain duration, n (%)			
1 month to 6 months	1 (2.7)	0 (0)	1 (5)
6 months to 1 year	4 (11)	2 (12)	2 (10)
1 to 2 years	3 (8.1)	3 (18)	0 (0)
2 to 5 years	10 (27)	3 (18)	7 (35)
More than 5 years	19 (51)	9 (53)	10 (50)
Medication use, n (%)	15 (39)	10 (56)	5 (25)
Current sick leave, n (%)	7 (18)	4 (22)	3 (15)
Pain intensity (NRS^a^ 0‐10), mean (SD)	3.92 (2.05)	3.67 (2.00)	4.15 (2.11)
Oswestry Disability Index, mean (SD)	24.58 (9.57)	24.43 (10.32)	24.72 (9.11)

a NRS: numeric rating scale.

### Usability

The median score of the SUS among both groups was 82.5 (IQR 62.5‐91.9), which indicates a good usability [24]. The between-group comparison of medians revealed no differences (Physio+App: 77.5, IQR 58.1‐91.9; App: 86.3, IQR 65‐90.6; *P*=.57). Table 2 displays the frequency of each answer on the Likert scale per question for each group. The inspection of single items revealed that most patients (20/38, 53%) strongly agreed on item 3 (“I thought the system was easy to use.”). Furthermore, 24/38 (63%) strongly disagreed on item 4 (“I think that I would need the support of a technical person to be able to use this system.”).

**Table 2.** Frequency of answers in the System Usability Scale that evaluates the usability of a mobile app, specifically the app “NOLA” used in this trial, which combines exercise and education for patients with low back pain. Each item could be answered with a numeric value between 0 (“do not agree”) to 4 (“fully agree”). Between-group differences were evaluated using the Wilcoxon rank sum test.

		Between-group comparison		
Item^a^	Overall, N=38	App only, n=18	Physio+App, n=20	*P* value^b^
I think that I would like to use this system frequently, n (%)				.49
0	1 (2.6)	1 (5.6)	0 (0)	
1	4 (11)	1 (5.6)	3 (15)	
2	9 (24)	5 (28)	4 (20)	
3	12 (32)	7 (39)	5 (25)	
4	12 (32)	4 (22)	8 (40)	
I found the system unnecessarily complex, n (%)				.50
0	19 (50)	8 (44)	11 (55)	
1	6 (16)	3 (17)	3 (15)	
2	10 (26)	5 (28)	5 (25)	
3	2 (5.3)	2 (11)	0 (0)	
4	1 (2.6)	0 (0)	1 (5)	
I thought the system was easy to use, n (%)				.33
0	0 (0)	0 (0)	0 (0)	
1	1 (2.6)	1 (5.6)	0 (0)	
2	6 (16)	3 (17)	3 (15)	
3	11 (29)	6 (33)	5 (25)	
4	20 (53)	8 (44)	12 (60)	
I think that I would need the support of a technical person to be able to use this system, n (%)				.33
0	24 (63)	10 (56)	14 (70)	
1	5 (13)	3 (17)	2 (10)	
2	7 (18)	3 (17)	4 (20)	
3	1 (2.6)	1 (5.6)	0 (0)	
4	1 (2.6)	1 (5.6)	0 (0)	
I found the various functions in this system were well integrated, n (%)				.69
0	0 (0)	0 (0)	0 (0)	
1	0 (0)	0 (0)	0 (0)	
2	9 (24)	5 (28)	4 (20)	
3	18 (47)	8 (44)	10 (50)	
4	11 (29)	5 (28)	6 (30)	
I thought there was too much inconsistency in this system, n (%)				.59
0	14 (37)	6 (33)	8 (40)	
1	12 (32)	8 (44)	4 (20)	
2	9 (24)	4 (22)	5 (25)	
3	3 (7.9)	0 (0)	3 (15)	
4	0 (0)	0 (0)	0 (0)	
I would imagine that most people would learn to use this system very quickly, n (%)				.37
0	0 (0)	0 (0)	0 (0)	
1	1 (2.6)	1 (5.6)	0 (0)	
2	5 (13)	2 (11)	3 (15)	
3	16 (42)	9 (50)	7 (35)	
4	16 (42)	6 (33)	10 (50)	
I found the system very cumbersome to use, n (%)				.35
0	18 (47)	7 (39)	11 (55)	
1	13 (34)	7 (39)	6 (30)	
2	5 (13)	3 (17)	2 (10)	
3	2 (5.3)	1 (5.6)	1 (5)	
4	0 (0)	0 (0)	0 (0)	
I felt very confident using the system, n (%)				.51
0	1 (2.6)	0 (0)	1 (5)	
1	3 (7.9)	3 (17)	0 (0)	
2	6 (16)	2 (11)	4 (20)	
3	13 (34)	7 (39)	6 (30)	
4	15 (39)	6 (33)	9 (45)	
I needed to learn a lot of things before I could get going with this system, n (%)				.96
0	17 (45)	8 (44)	9 (45)	
1	9 (24)	4 (22)	5 (25)	
2	6 (16)	4 (22)	2 (10)	
3	6 (16)	2 (11)	4 (20)	
4	0 (0)	0 (0)	0 (0)	

a 0 = do not agree to 4=fully agree.

b Wilcoxon rank sum test.

### Perceptions Toward Telemedicine

The median overall score in the TMPQ (0‐60) was 45 (IQR 36.3‐53.8) in the Physio+App group and 43.5 (IQR 36.5‐50.5) in the App group (*P*=.25; Multimedia Appendix 3). Simple linear regression with within-group change of pain intensity (numeric rating scale) as an independent variable revealed a positive association with the TMPQ score (*F*_1,35_=7.5, *P*=.01), but change of pain intensity could only explain 15.4% of the variance in the TMPQ score.

**Figure 3.**
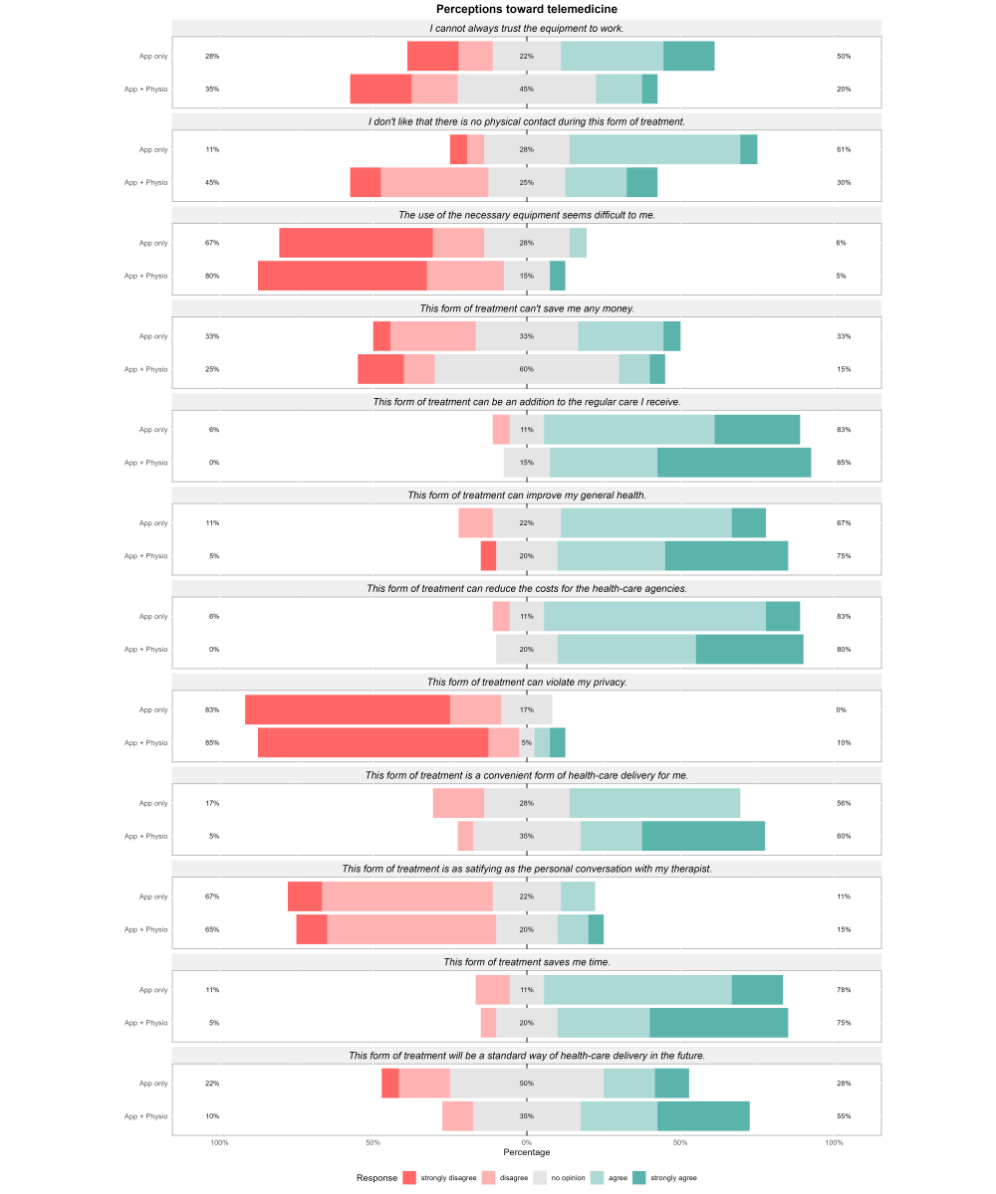
Answers in the Telemedicine Perception Questionnaire per group. The questionnaire evaluates the perceptions of an individual toward telemedicine in general. The questionnaire was adapted to include only meaningful questions related to the intervention under evaluation in this trial. Participants could answer with “strongly disagree,” “disagree,” “no opinion,” “agree,” or “strongly agree” to each item. Answers are displayed as colored bars as well as percentages for each direction.

Figure 3 shows the frequency of each answer per question of the TMPQ. Participants in the Physio+App group were indecisive (“no opinion”: 9/20, 45%) about whether they could always trust the technical equipment to work or not, while 9/18 (50%) participants in the App group agreed or strongly agreed with that statement. Further, 16/20 (80%) participants in the Physio+App group and 12/18 (67%) in the App group did not find the use of the necessary equipment difficult. A total of 6/20 (30%) participants in the Physio+App group and 11/18 (61.6%) in the App group agreed or strongly agreed to the statement that they do not like that telemedicine does not include physical contact. In line with this, only 3/20 (15%) participants in the Physio+App group and 2/18 (11%) in the App group agreed that telemedicine is as satisfying as a personal conversation with the therapist. Despite this, most participants (Physio+App: 12/20, 60%; App: 10/18, 56%) agreed that telemedicine is a convenient form of health care delivery, and 17/20 (85%) participants in the Physio+App group and 15/18 (84%) in the App group rated telemedicine as an adequate addition to usual care. Regarding the advantages of telemedicine, most participants (Physio+App: 15/20, 75%; App: 12/18, 67%) think that telemedicine can improve the general health, reduce health care costs (Physio+App: 16/20, 80%; App: 15/18, 83%), and save time (Physio+App: 17/20, 75%; App: 14/18, 78%). No tendency was found regarding the reduction of personal costs in both groups. Most participants (Physio+App: 17/20, 85%; App: 15/18, 84%) were not concerned about a violation of their privacy through a telemedicine app. Finally, 11/20 (55%) participants in the Physio+App group, but only 5/18 (28%) in the App group, agreed on the statement that telemedicine will be a standard way of health care delivery in the future.

### App Usage, Adherence, and Motivation

Thirty-seven participants self-reported their app usage. In the App group, 15/18 (84%) participants, and in the Physio+App group, 17/19 (90%) participants reached the goal of using the app at least 2 times per week (Table 3). Participants in the Physio+App group attended, on average, 5.3 (SD 1.2) sessions of physiotherapy.

**Table 3.** Adherence, app usage, and motivation to use the app “NOLA” in the context of the randomized controlled trial. As not all participants answered all questions, the number of valid answers per question is displayed.

Variable	Valid answers	App only, n=18	Physio+App, n=20	*P* Value^a^
Adherence, n (%)	38			.16
I was able to use the app as planned for 6 weeks at a time		7 (39)	12 (60)	
I had to interrupt app use due to illness		5 (28)	4 (20)	
I had to interrupt app use due to holidays		4 (22)	0 (0)	
I had to interrupt app use due to scheduling problems		3 (17)	0 (0)	
I had to interrupt app use due to an exacerbation of pain in my back		1 (5.6)	0 (0)	
I had to interrupt app use because of pain in another part of my body		0 (0)	2 (10)	
I had to interrupt app use due to other reasons		3 (17)	4 (20)	
How often have you trained your personal workout (back) with the app? n (%)	37			.25
Less than 2 days per week		3 (17)	2 (11)	
2 days per week		5 (28)	2 (11)	
3‐5 days per week		7 (39)	9 (47)	
Nearly every day		3 (17)	6 (32)	
Think about the last 6 weeks: How motivated were you to open the app independently and train with it? n (%)	33			.80
I was not motivated at all, but thought of the results of the study		2 (13)	0 (0)	
I have found it very difficult to open the app regularly		0 (0)	0 (0)	
It took me a little effort to open the app at times		3 (19)	3 (18)	
I was mostly motivated		7 (44)	14 (82)	
I was always highly motivated to do the training frequently and regularly		4 (25)	0 (0)	
How would you rate the usefulness of the NOLA app in school grades? n (%)	35			.23
1		2 (13)	6 (32)	
2		10 (63)	10 (53)	
3		3 (19)	2 (11)	
4		1 (6.3)	0 (0)	
5		0 (0)	0 (0)	
6		0 (0)	1 (5.3)	
How strong is your desire to continue training with the app? n (%)	38			.23
No more need		2 (11)	0 (0)	
I’m still thinking about it, but the trend is more towards habit again (gym, sports club, rehab sports).		2 (11)	3 (15)	
I am still indecisive		7 (39)	6 (30)	
I would give it a chance, even if the app is chargeable		5 (28)	6 (30)	
I am very enthusiastic. I will definitely continue to use the app, as I see great progress		2 (11)	5 (25)	

a Wilcoxon rank sum test.

Of 38 participants, 12/20 (60%) in the Physio+App group and 7/18 (39%) in the App group indicated that they were able to use the app as planned for 6 weeks at a time, showing a higher compliance in the Physio+App group. The reasons for an interruption of the app use are displayed in Table 3.

Most participants (Physio+App: 14/16, 82%; App: 7/17, 44%) indicated they were mostly motivated to train with the app, while the motivation was higher in the Physio+App group. Although the usefulness of the app was rated in both groups with a median of 2 (median absolute deviation: 0, n=35), 6/20 (30%) participants in the Physio+App group and 7/18 (39%) in the App group were indecisive about the desire to train with the app in the future (Table 3).

### Drop-Out Analysis

The group of participants who dropped out after randomization (n=26; 40%) showed statistically significant lower pain (dropouts: mean 3.0, SD 2.0, completers: mean 3.9, SD 2.0, *P*=.02) values at baseline compared to those who completed the intervention (n=38, 60%), but did not differ in disability and age (Table 4). From those participants who dropped out of the study (n=26), only 5 reported the reasons. Two participants dropped out for family reasons, 1 had an accident, 1 participant from the Physio+App group had no time for the physiotherapy sessions, and 1 indicated that the usage of the app was too complicated.

**Table 4.** Comparison of baseline values between those who dropped out and those who completed the intervention.

Variable	Dropouts (n=26)	Completers (n=38)	*P* value^a^
Age, mean (SD)	52.9 (12.0)	49.9 (13.5)	.35
Pain, mean (SD)	3.0 (2.0)	3.9 (2.0)	.02
Disability, mean (SD)	19.0 (9.4)	24.6 (9.6)	.09

a Wilcoxon rank sum test.

## Discussion

### Principal Findings

This preplanned secondary analysis shows that an app that delivered an exercise intervention with education showed good usability. It was also shown that participants with LBP have a generally positive perception of telemedicine, but it should be used as an adjunct to in-person therapy with physical contact. Although there was a tendency for participants who received the app as an adjunct to standard physiotherapy to perceive usability and telemedicine in general as better, there was no clear difference between the 2 groups. Motivation to use the app during the intervention phase was mostly high, with most patients using the app on average 3 to 5 days per week. However, most patients were indecisive about using the app in the future. Only a few patients reported the reasons for a dropout from the study, which might bias the results.

While several studies investigated patients’ perceptions toward a specific telemedicine modality, the perception toward telemedicine in general of patients with LBP has not yet been evaluated extensively. Our study adds this knowledge about perceptions toward telemedicine in general after using a telemedicine approach alone and in addition to in-person physiotherapy. Barton et al [13] evaluated patients’ perceptions regarding the use of physiotherapy via telemedicine approaches during the COVID-19 pandemic in patients with musculoskeletal pain. The results indicated that patients could accept telemedicine when there is an option to have regular in-person appointments with the therapist. This is in line with results from the TMPQ in our study, which showed that although participants were, in general, open toward telemedicine, they were missing the physical contact with a therapist. Similar findings are identifiable from a systematic review of qualitative studies for the population of chronic pain regarding perceptions toward a specific telemedicine intervention [10]. Our data showed that participants who received only the app intervention were more likely to agree that they missed the physical contact in telemedicine interventions. Having physical contact regularly is also a critical facilitator for therapists [25]. Participants in our study believed that telemedicine can reduce health care costs and save time. This is in line with findings from qualitative studies on user experiences of telemedicine interventions in patients with LBP [26,27] and musculoskeletal pain [13].

Two qualitative studies showed that technical issues with an app-based intervention for LBP are an important factor that negatively influences the user experience [26,28]. The good usability rated in the SUS indicates that participants in our analysis did not have relevant technical issues, which could partly explain why perceptions toward telemedicine were mainly positive. Further, participants who received physiotherapy in addition to the app intervention, and thus had technical support throughout the whole intervention, trusted the equipment to work more than those who received only the app intervention. The usefulness of the app was rated with a median of 2 (IQR 2 - 2, on a scale from 1 to 6, with 1 being the best), which leads to the conclusion that the use of the app was well accepted among the participants who completed the intervention. This is in line with findings from Hasenöhrl et al [29] who evaluated the acceptance of therapeutic exercises delivered via an app.

Svendsen et al [26] found that decreased or increased pain could lead to not using the app as often as planned. In line with this, our dropout analysis showed that participants who dropped out showed lower levels of pain compared to those who completed. It can be assumed that participants dropped out because the burden of their LBP was not high enough to continue with the intervention.

Our study shows some limitations that must be considered. The dropout rate was relatively high in our study, but only a few patients reported reasons for dropout. It cannot be ruled out that some participants dropped out due to dissatisfaction with the app, which could bias results and does not allow for the clear conclusion that acceptability was high. We evaluated patients’ perceptions with questionnaires and not with interviews, which could give a better insight into the user experience. As outcome measurements from the primary analysis were already time-consuming, we did not perform additional interviews in order to keep the motivation to participate in follow-up measurements high. Although the sample was relatively small, the included patients showed an age range where the prevalence of back pain is at its highest [30]. Regarding the adherence to the intervention and the app usage, we were not able to retrieve usage data directly from the app due to data protection reasons. As a consequence, we had to rely on self-reported data about adherence and app usage. It is known from other medical conditions that self-reported measures of adherence may overestimate the real effect [31].

The results of our study have implications for clinical practice. Patients seem to accept telemedicine approaches in general, but their use in combination with an in-person setting should be considered. Furthermore, it is important to ensure that technical issues are reduced and technical support is available to enhance the user experience and thus increase patient satisfaction.

Future research should focus on patients’ perceptions regarding different delivery pathways, such as apps, websites, or videoconferencing, in order to obtain knowledge about preferences regarding specific forms of treatment. Additionally, it has to be investigated which factors influence adherence to telemedicine in a real-life setting, as well as which subgroups may show a higher acceptance and could thus benefit more from telemedicine interventions.

### Conclusions

Our randomized controlled trial indicated good usability of the app “NOLA” regarding need for support, complexity, and training, as well as positive patient perceptions toward telemedicine in general in those patients who completed the intervention. This was independent of whether participants received only the app intervention or physiotherapy in addition to the app intervention. Despite concerns about the lack of physical contact, participants recognized telemedicine as a convenient addition to usual care. App usage and participant motivation were high, emphasizing the potential of such interventions in managing LBP. However, it has to be kept in mind that the high dropout rate might have been due to dissatisfaction with the intervention, which could bias our results. The findings from our study contribute insights to the evolving field of telemedicine, suggesting that well-designed apps could enhance patient engagement and satisfaction in patients with LBP. Further exploration and optimization of app-based interventions are needed for the effective integration into routine clinical practice.

## Supplementary material

10.2196/59866 Multimedia Appendix 1 System Usability Scale - German adapted version.

10.2196/59866 Multimedia Appendix 2 TMPQ – Telemedicine Perception Questionnaire – German adapted version. TMPQ: Telemedicine Perception Questionnaire.

10.2196/59866 Multimedia Appendix 3 Answers in the Telemedicine Perception Questionnaire per item.

10.2196/59866 Checklist 1 CONSORT-eHealth checklist (V 1.6.1).

## Abbreviations

CONSORT: Consolidated Standards of Reporting Trials
*ICD-10*: *International Statistical Classification of Diseases, Tenth Revision*
LBP: low back pain
SUS: System Usability Scale
TMPQ: Telemedicine Perception Questionnaire
