# Supplementary material for: Usability and Acceptability of an App-Based Approach to Treat Low Back Pain: Preplanned Secondary Analysis of a Randomized Controlled Trial
Source: JMIR Form Res. 2025 Aug 25;9:e59866. doi: 10.2196/59866 (PMC12377697; doi:10.2196/59866)
Supplement: Multimedia Appendix 1 — System Usability Scale - German adapted version. [file formative-v9-e59866-s001.docx]

Bitte beurteilen Sie auf der folgenden Skala, inwieweit Sie den Aussagen zustimmen.

1. Ich denke ich würde die App regelmäßig nutzen.

| lehne völlig ab |  |  |  | stimme völlig zu |
| --- | --- | --- | --- | --- |
|  |  |  |  |  |
| 0 | 1 | 2 | 3 | 4 |

1. Die App erscheint mir unnötig kompliziert.

| lehne völlig ab |  |  |  | stimme völlig zu |
| --- | --- | --- | --- | --- |
|  |  |  |  |  |
| 0 | 1 | 2 | 3 | 4 |

1. Ich finde, die App ist einfach zu benutzen.

| lehne völlig ab |  |  |  | stimme völlig zu |
| --- | --- | --- | --- | --- |
|  |  |  |  |  |
| 0 | 1 | 2 | 3 | 4 |

1. Ich denke, ich bräuchte technische Unterstützung um die App nutzen zu können.

| lehne völlig ab |  |  |  | stimme völlig zu |
| --- | --- | --- | --- | --- |
|  |  |  |  |  |
| 0 | 1 | 2 | 3 | 4 |

1. Ich finde, dass die verschiedenen Funktionen der App gut integriert sind.

| lehne völlig ab |  |  |  | stimme völlig zu |
| --- | --- | --- | --- | --- |
|  |  |  |  |  |
| 0 | 1 | 2 | 3 | 4 |

1. Die App erscheint mir zu uneinheitlich.

| lehne völlig ab |  |  |  | stimme völlig zu |
| --- | --- | --- | --- | --- |
|  |  |  |  |  |
| 0 | 1 | 2 | 3 | 4 |

1. Ich glaube, dass die meisten Leute die Benutzung der App schnell erlernen können.

| lehne völlig ab |  |  |  | stimme völlig zu |
| --- | --- | --- | --- | --- |
|  |  |  |  |  |
| 0 | 1 | 2 | 3 | 4 |

1. Die App erscheint mir sehr umständlich zu benutzen.

| lehne völlig ab |  |  |  | stimme völlig zu |
| --- | --- | --- | --- | --- |
|  |  |  |  |  |
| 0 | 1 | 2 | 3 | 4 |

1. Ich fühle mich bei der Benutzung der App sehr sicher.

| lehne völlig ab |  |  |  | stimme völlig zu |
| --- | --- | --- | --- | --- |
|  |  |  |  |  |
| 0 | 1 | 2 | 3 | 4 |

1. Ich musste einiges lernen, um mit der App zurecht zu kommen.

| lehne völlig ab |  |  |  | stimme völlig zu |
| --- | --- | --- | --- | --- |
|  |  |  |  |  |
| 0 | 1 | 2 | 3 | 4 |
