# Supplementary material for: Usability and Acceptability of an App-Based Approach to Treat Low Back Pain: Preplanned Secondary Analysis of a Randomized Controlled Trial
Source: JMIR Form Res. 2025 Aug 25;9:e59866. doi: 10.2196/59866 (PMC12377697; doi:10.2196/59866)
Supplement: Multimedia Appendix 2 — TMPQ – Telemedicine Perception Questionnaire – German adapted version. TMPQ: Telemedicine Perception Questionnaire. [file formative-v9-e59866-s002.docx]

Original work:

## Demiris G, Speedie S, Finkelstein S. A questionnaire for the assessment of patients' impressions of the risks and benefits of home telecare. J Telemed Telecare. 2000;6(5):278-84. doi: 10.1258/1357633001935914. PMID: 11070589.

German validation:

Altmann P, Ivkic D, Ponleitner M, Leutmezer F, Willinger U, Schmoeger M, Berger T, Bsteh G, Löffler-Stastka H. Individual Perception of Telehealth: Validation of a German Translation of the Telemedicine Perception Questionnaire and a Derived Short Version. Int J Environ Res Public Health. 2022 Jan 14;19(2):902. doi: 10.3390/ijerph19020902. PMID: 35055724; PMCID: PMC8775421.

# 17 items, 5 Antwortoptionen

**5** stimme sehr zu

**4** stimme zu

**3** neutral / ich habe keine Meinung

**2** stimme nicht zu

**1** stimme überhaupt nicht zu

# Negativ formulierte Items sind gegensätzlich gewichtet

# Diese Behandlungsform kann meine Privatsphäre verletzen.

1. stimme sehr zu
2. stimme zu
3. neutral / ich habe keine Meinung
4. stimme nicht zu
5. stimme überhaupt nicht zu

# Der Einsatz der notwendigen Ausrüstung erscheint mir schwierig.

1. stimme sehr zu
2. stimme zu
3. neutral / ich habe keine Meinung
4. stimme nicht zu
5. stimme überhaupt nicht zu

# Diese Methode zur Behandlung mit meinem Therapeuten/meiner Therapeutin ist genauso zufriedenstellend wie das persönliche Gespräch.

**5** stimme sehr zu

**4** stimme zu

**3** neutral / ich habe keine Meinung

**2** stimme nicht zu

**1** stimme überhaupt nicht zu

# Diese Behandlungsform kann meine allgemeine Gesundheit verbessern.

**5** stimme sehr zu

**4** stimme zu

**3** neutral / ich habe keine Meinung

**2** stimme nicht zu

**1** stimme überhaupt nicht zu

# Diese Behandlungsform kann mir kein Geld ersparen.

1. stimme sehr zu
2. stimme zu
3. neutral / ich habe keine Meinung
4. stimme nicht zu
5. stimme überhaupt nicht zu

# Mir gefällt es nicht, dass bei dieser Behandlungsform kein physischer Kontakt zu meinem Therapeuten/zu meiner Therapeutin besteht.

**5** stimme sehr zu

**4** stimme zu

**3** neutral / ich habe keine Meinung

**2** stimme nicht zu

**1** stimme überhaupt nicht zu

# Diese Behandlungsform ist für mich eine bequeme Form für medizinische Versorgung in der Zukunft.

**5** stimme sehr zu

**4** stimme zu

**3** neutral / ich habe keine Meinung

**2** stimme nicht zu

**1** stimme überhaupt nicht zu

# Diese Behandlungsform spart mir Zeit.

**5** stimme sehr zu

**4** stimme zu

**3** neutral / ich habe keine Meinung

**2** stimme nicht zu

**1** stimme überhaupt nicht zu

# Diese Behandlungsform wird in der Zukunft ein Standard für medizinische Versorgung sein.

**5** stimme sehr zu

**4** stimme zu

**3** neutral / ich habe keine Meinung

**2** stimme nicht zu

**1** stimme überhaupt nicht zu

# Diese Behandlungsform kann eine Ergänzung zu meiner regulären Versorgung sein.

**5** stimme sehr zu

**4** stimme zu

**3** neutral / ich habe keine Meinung

**2** stimme nicht zu

**1** stimme überhaupt nicht zu

# Diese Behandlungsform kann die Kosten der Kranken- und Sozialversicherungen senken.

**5** stimme sehr zu

**4** stimme zu

**3** neutral / ich habe keine Meinung

**2** stimme nicht zu

**1** stimme überhaupt nicht zu

# Ich kann nicht immer darauf vertrauen, dass die Ausrüstung funktioniert.

1. stimme sehr zu
2. stimme zu
3. neutral / ich habe keine Meinung
4. stimme nicht zu
5. stimme überhaupt nicht zu
