# Supplementary material for: Usability and Acceptability of an App-Based Approach to Treat Low Back Pain: Preplanned Secondary Analysis of a Randomized Controlled Trial
Source: JMIR Form Res. 2025 Aug 25;9:e59866. doi: 10.2196/59866 (PMC12377697; doi:10.2196/59866)
Supplement: Multimedia Appendix 3 — Answers in the Telemedicine Perception Questionnaire per item. [file formative-v9-e59866-s003.docx]

| **Item** | **Overall**, N = 38 | **App only**, N = 18 | **Physio+App**, N = 20 | **p-value***^1^* |
| --- | --- | --- | --- | --- |
| **1. This form of treatment can violate my privacy., N (%)** |  |  |  | 0.7 |
| strongly disagree | 27 (71%) | 12 (67%) | 15 (75%) |  |
| disagree | 5 (13%) | 3 (17%) | 2 (10%) |  |
| no opinion | 4 (11%) | 3 (17%) | 1 (5.0%) |  |
| agree | 1 (2.6%) | 0 (0%) | 1 (5.0%) |  |
| strongly agree | 1 (2.6%) | 0 (0%) | 1 (5.0%) |  |
| **2. The use of the necessary equipment seems difficult to me., N (%)** |  |  |  | 0.6 |
| strongly disagree | 20 (53%) | 9 (50%) | 11 (55%) |  |
| disagree | 8 (21%) | 3 (17%) | 5 (25%) |  |
| no opinion | 8 (21%) | 5 (28%) | 3 (15%) |  |
| agree | 1 (2.6%) | 1 (5.6%) | 0 (0%) |  |
| strongly agree | 1 (2.6%) | 0 (0%) | 1 (5.0%) |  |
| **3. This form of treatment is as satifying as the personal conversation with my therapist., N (%)** |  |  |  | 0.8 |
| strongly disagree | 4 (11%) | 2 (11%) | 2 (10%) |  |
| disagree | 21 (55%) | 10 (56%) | 11 (55%) |  |
| no opinion | 8 (21%) | 4 (22%) | 4 (20%) |  |
| agree | 4 (11%) | 2 (11%) | 2 (10%) |  |
| strongly agree | 1 (2.6%) | 0 (0%) | 1 (5.0%) |  |
| **4. This form of treatment can improve my general health., N (%)** |  |  |  | 0.13 |
| strongly disagree | 1 (2.6%) | 0 (0%) | 1 (5.0%) |  |
| disagree | 2 (5.3%) | 2 (11%) | 0 (0%) |  |
| no opinion | 8 (21%) | 4 (22%) | 4 (20%) |  |
| agree | 17 (45%) | 10 (56%) | 7 (35%) |  |
| strongly agree | 10 (26%) | 2 (11%) | 8 (40%) |  |
| **5. This form of treatment can't save me any money., N (%)** |  |  |  | 0.6 |
| strongly disagree | 4 (11%) | 1 (5.6%) | 3 (15%) |  |
| disagree | 7 (18%) | 5 (28%) | 2 (10%) |  |
| no opinion | 18 (47%) | 6 (33%) | 12 (60%) |  |
| agree | 7 (18%) | 5 (28%) | 2 (10%) |  |
| strongly agree | 2 (5.3%) | 1 (5.6%) | 1 (5.0%) |  |
| **6. I don't like that there is no physical contact during this form of treatment., N (%)** |  |  |  | 0.057 |
| strongly disagree | 3 (7.9%) | 1 (5.6%) | 2 (10%) |  |
| disagree | 8 (21%) | 1 (5.6%) | 7 (35%) |  |
| no opinion | 10 (26%) | 5 (28%) | 5 (25%) |  |
| agree | 14 (37%) | 10 (56%) | 4 (20%) |  |
| strongly agree | 3 (7.9%) | 1 (5.6%) | 2 (10%) |  |
| **7. This form of treatment is a convenient form of health-care delivery for me., N (%)** |  |  |  | 0.089 |
| strongly disagree | 0 (0%) | 0 (0%) | 0 (0%) |  |
| disagree | 4 (11%) | 3 (17%) | 1 (5.0%) |  |
| no opinion | 12 (32%) | 5 (28%) | 7 (35%) |  |
| agree | 14 (37%) | 10 (56%) | 4 (20%) |  |
| strongly agree | 8 (21%) | 0 (0%) | 8 (40%) |  |
| **8. This form of treatment saves me time., N (%)** |  |  |  | 0.2 |
| strongly disagree | 0 (0%) | 0 (0%) | 0 (0%) |  |
| disagree | 3 (7.9%) | 2 (11%) | 1 (5.0%) |  |
| no opinion | 6 (16%) | 2 (11%) | 4 (20%) |  |
| agree | 17 (45%) | 11 (61%) | 6 (30%) |  |
| strongly agree | 12 (32%) | 3 (17%) | 9 (45%) |  |
| **9. This form of treatment will be a standard way of health-care delivery in the future., N (%)** |  |  |  | 0.072 |
| strongly disagree | 1 (2.6%) | 1 (5.6%) | 0 (0%) |  |
| disagree | 5 (13%) | 3 (17%) | 2 (10%) |  |
| no opinion | 16 (42%) | 9 (50%) | 7 (35%) |  |
| agree | 8 (21%) | 3 (17%) | 5 (25%) |  |
| strongly agree | 8 (21%) | 2 (11%) | 6 (30%) |  |
| **10. This form of treatment can be an addition to the regular care I receive., N (%)** |  |  |  | 0.2 |
| strongly disagree | 0 (0%) | 0 (0%) | 0 (0%) |  |
| disagree | 1 (2.6%) | 1 (5.6%) | 0 (0%) |  |
| no opinion | 5 (13%) | 2 (11%) | 3 (15%) |  |
| agree | 17 (45%) | 10 (56%) | 7 (35%) |  |
| strongly agree | 15 (39%) | 5 (28%) | 10 (50%) |  |
| **11. This form of treatment can reduce the costs for the health-care agencies., N (%)** |  |  |  | 0.3 |
| strongly disagree | 0 (0%) | 0 (0%) | 0 (0%) |  |
| disagree | 1 (2.6%) | 1 (5.6%) | 0 (0%) |  |
| no opinion | 6 (16%) | 2 (11%) | 4 (20%) |  |
| agree | 22 (58%) | 13 (72%) | 9 (45%) |  |
| strongly agree | 9 (24%) | 2 (11%) | 7 (35%) |  |
| **12. I cannot always trust the equipment to work., N (%)** |  |  |  | 0.2 |
| strongly disagree | 7 (18%) | 3 (17%) | 4 (20%) |  |
| disagree | 5 (13%) | 2 (11%) | 3 (15%) |  |
| no opinion | 13 (34%) | 4 (22%) | 9 (45%) |  |
| agree | 9 (24%) | 6 (33%) | 3 (15%) |  |
| strongly agree | 4 (11%) | 3 (17%) | 1 (5.0%) |  |
| *^1^*Wilcoxon rank sum test | | | | |
